# The largest esophageal foreign body in adults: A case report

**DOI:** 10.1016/j.amsu.2020.04.039

**Published:** 2020-05-01

**Authors:** Firas Shaker Mahmoud Al-Faham, Samer Makki Mohamed Al-Hakkak

**Affiliations:** a Department of Surgery, College of Medicine, Kerbala University, Kerbala City, Iraq; b Department of Surgery, Faculty of Medicine, Jabir Ibn Hayyan Medical University, Najaf City, Iraq

**Keywords:** Esophagus, Foreign body, Adult, Self-injurious behavior, Psychiatric patient, FB, Foreign body

## Abstract

**Introduction:**

The swallowing of foreign bodies can be accidental or intentional. The majority of the cases of accidental foreign body ingestion are observed in children. In adults, foreign body ingestion can be accidental, related to specific pathological changes of the digestive tract, or deliberate, as observed in patients with psychiatric diseases or in those released from the prison.

**Case presentation:**

A 42-year-old male was admitted to the emergency department with symptoms including choking, drooling from the mouth, holding his neck, and aphonia. He had a history of psychiatric illness with suicidal ingestion of a foreign body. After stabilization, he was sent for chest radiograph, which revealed a significant radiopaque shadow the shape of a spanner, occupying the whole length of the esophagus. Emergency rigid esophagoscopy was performed to save the patient's life.

**Discussion:**

The patient swallowed the largest hard foreign body to harm himself or his family, to get the attention of his family, or as a suicide attempt. Such patients require urgent intervention by rigid esophagoscopy to reduce the risk of complications and to save the patients’ lives. Further follow-up is essential due to the possibility of repeated foreign body ingestion.

**Conclusion:**

While taking care of psychiatric patients, close observation by family members is mandatory to prevent them from harming themselves and to prevent suicide attempts by swallowing sharp, hard, large, and dangerous foreign bodies such as the size 17 wrench spanner observed in the present case.

## Highlights

• An impacted oesophagal foreign body is a top emergency.
• First-line management of stuck foreign bodies in the esophagus is by endoscopy.
• Psychological upset patient may swallow dangerous large objects.

## 1 Introduction

Foreign body (FB) ingestion is observed most frequently in the pediatric age group. Its incidence peaks between the ages of approximately six months and six years and it is primarily accidental [1]. However, in adults, it is more likely to represent self-harm. It is observed in patients with psychiatric disorders [2,3] and is reportedly overrepresented in prison settings [4]. Most of the ingested foreign bodies (80%–90%) pass spontaneously. Approximately 10%–20% of the cases of foreign body ingestion require endoscopic removal, while less than 1% require surgery for foreign body extraction or to treat the complications [5]. Although the majority of the FBs are benign and pass smoothly, FB ingestion is associated with increased morbidity. Particularly, care of patients who engage in repeated acts of deliberate self-harm can prove costly economically as well as in terms of the morale of their care providers. Intentional FB ingestion is often a recurring act to inflict self-harm or a part of attention-seeking behavior. Deliberate ingestion of FBs has relatively little representation in the medical literature, but is commonly reported by specialists in emergency medicine, general surgery, and gastroenterology [[6], [7], [8], [9]]. FB impaction frequently occurs at the level of normal esophageal physiologic narrowing. Previous upper-gastrointestinal surgery, congenital gut malformations, esophageal motility disorders, or eosinophilic esophagitis may also represent significant risk factors. Large FBs and FBs with irregular size may cause compression of the esophageal wall, edema, ischemia, and mucosal erosion. Esophageal perforation is a severe life-threatening complication and the risk of perforation is increased by the impaction of sharp or pointed objects and animal or fish bones [4,10]. The present case has been reported in line with the SCARE criteria [13].

## 2 Case presentation

**Fig. 1:**
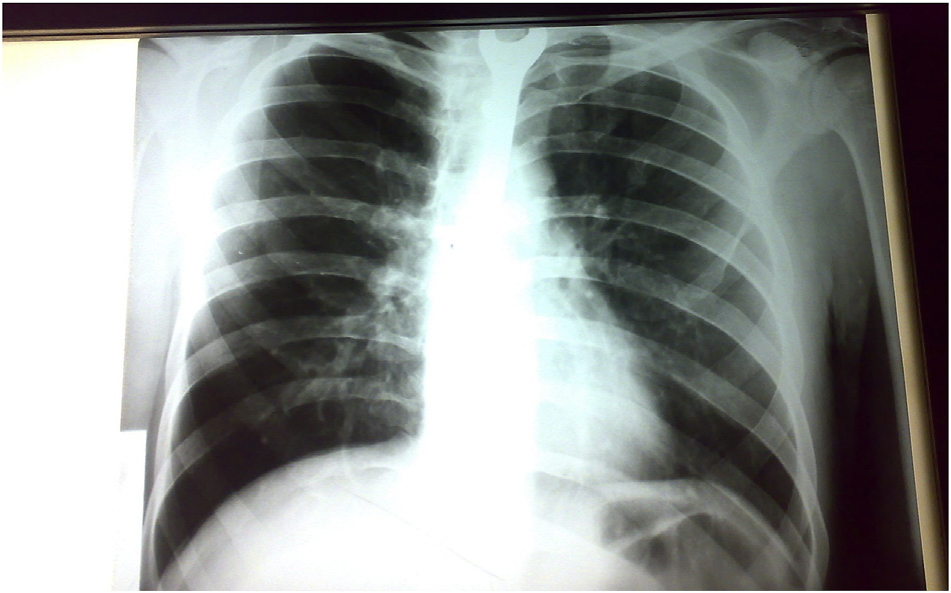
CXR show FB Completely impacted in the esophagus.

**Fig. 2:**
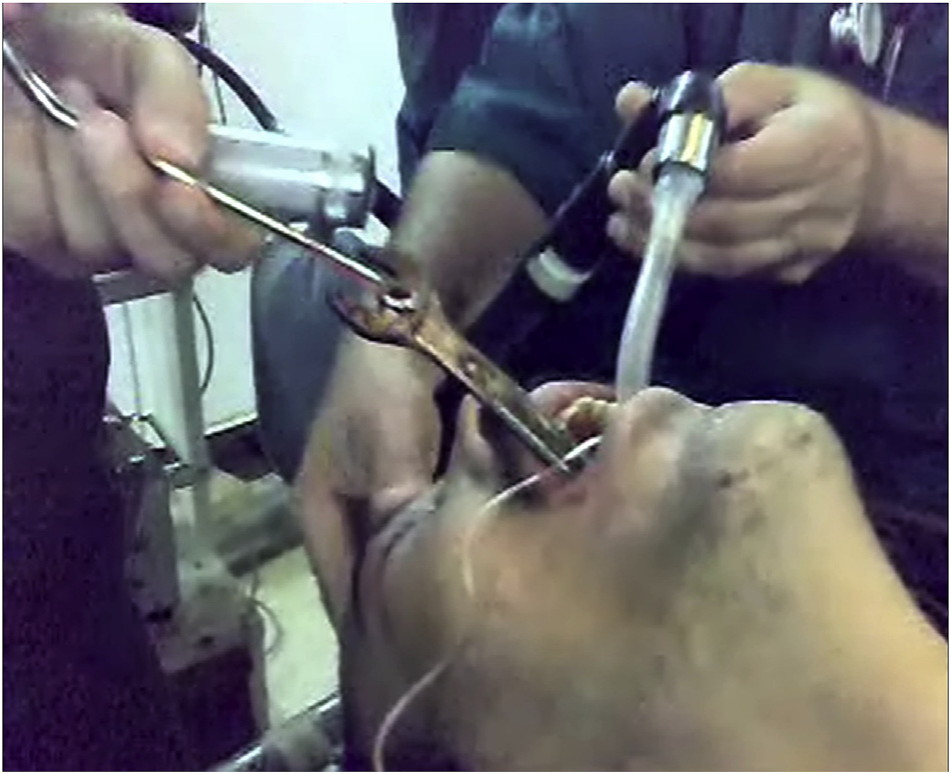
Magill forceps pull spanner from the mouth.

**Fig. 3:**
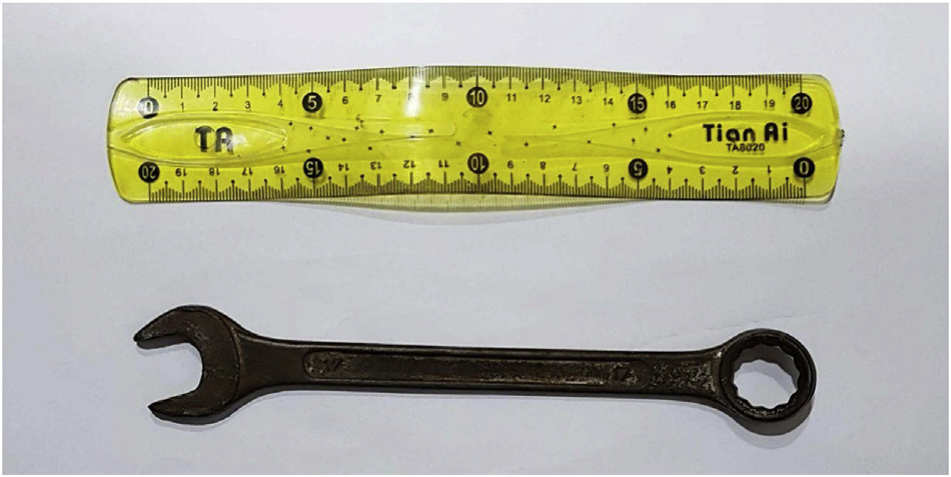
Real spanner gauge 17.

A 42-year-old man presented to the emergency department with symptoms including choking, drooling from the mouth, holding his neck with his hand, and aphonia. His family revealed that the patient had psychological abnormalities and depression and he had attempted suicide with swallowing a metallic object. The patient was hemodynamically stable. The arterial blood pressure was 130/80 mmHg and the pulse rate was 88 beats per minute. He was sent for chest, anterior-posterior, and lateral view radiographs that showed an abnormal radiopaque FB inside the esophagus and a shadow in the middle of the chest radiograph with a part of it obliterated by the heart and the vertebral shadow (Fig. 1). The FB had a shape similar to a spanner. Before the endoscopic intervention, the patient could not talk. His family gave an unclear history of the nature of his psychological problem. The patient was prepared for emergency surgery. He was admitted to the operation theater under general anesthesia and endotracheal intubation and rigid esophagoscopy was performed. The mouth was opened using a laryngoscope and blood and saliva were aspirated from the oropharynx. After passing a rigid esophagoscope through the orifice of the esophagus, we observed the open end of a spanner (wrench) that appeared stuck in the esophagus. After aspiration of blood and saliva around this part of the spanner, it was held with forceps and drawn out forcefully with the esophagoscope. After it reached the mouth, it was pulled out with Magill forceps (Fig. 2, Fig. 3). The esophagus was checked again using esophagoscopy for signs of injury. The esophagus was intact with no signs of perforation. After discontinuation of anesthesia, the patient was extubated and kept under observation. Subsequently, he was discharged without any complications. After surgery, he denied having any psychological problems, but was unable to sleep and showed irritability and restlessness. He also denied having any auditory or visual hallucinations. We advised him to visit the psychology department for a consultation, but he refused and was discharged from the hospital with his relatives.

## 3 Discussion

**Fig. 4:**
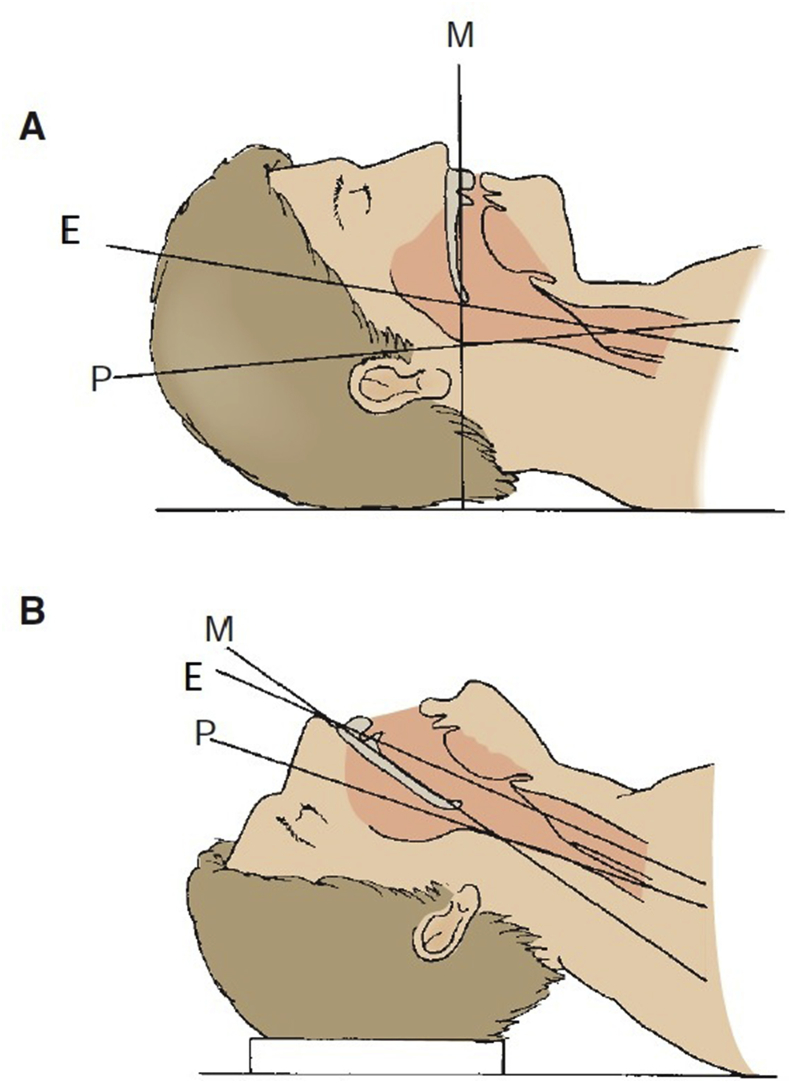
Axes of neck M mouth E Esophagus P Pharynx.

**Fig. 5:**
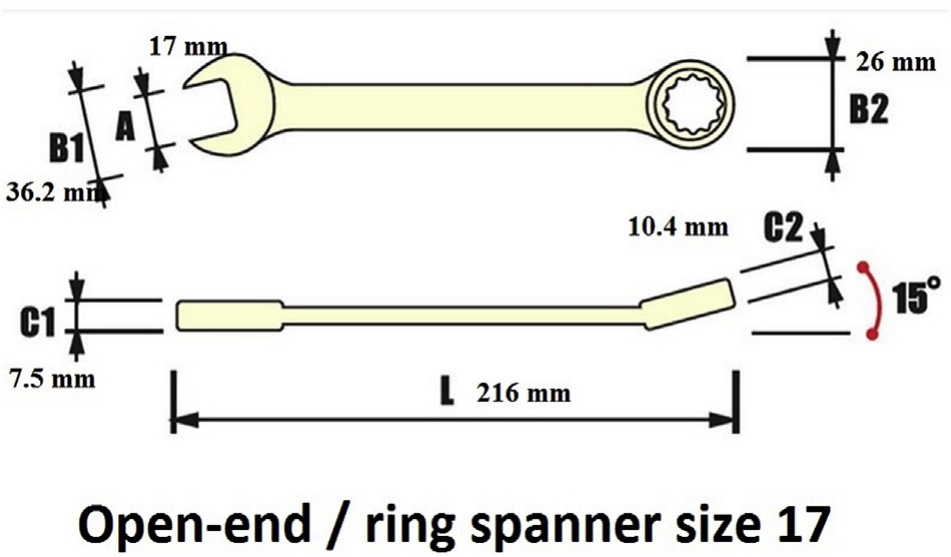
Spanner dimensions.

To the best of our knowledge, the present case describes the largest FB impacted in the esophagus of an adult patient. Acute esophageal FB impaction is believed to be the third most common non-biliary digestive endoscopic emergency after upper and lower gastrointestinal bleeding [11]. In adults, the esophagus is a muscular tube approximately ten inches in length (25 cm). It extends from the pharynx to the stomach with three sites of esophageal narrowing that typically offer resistance to FB ingestion. The first narrowing is at 7.2 inches (18 cm), at the beginning of the esophagus behind the cricoid cartilage. The second narrowing is at 11.2 inches (28 cm), at the point where the left bronchus and the arch of aorta cross the front of the throat. The third narrowing is at 17.2 inches (44 cm) where the esophagus enters the stomach. With the head in the neutral position, axis of the mouth (M), axis of the esophagus (E), and axis of the pharynx (P) are not aligned with one another. If the head is extended fully at the atlanto-occipital joint, the axis of the mouth is correctly placed. When the back of the head is raised off the table with a pillow, flexing the cervical vertebral column, the axes of the esophagus and the pharynx are aligned with the axis of the mouth (Fig. 4). Thus, it is very difficult to insert such a large foreign body inside the esophagus and how much time it needs! The literature on personality disorders covers a dazzling array of self-injurious behaviors including objects not only swallowed, but also inserted into the abdomen, heart, airway, bladder, breast, ear, legs, arms, and even into the skull [12]. Our case report reflects the characteristics of such self-injurious behavior. Internal feelings of emptiness and unbearable tension often precede such behaviour. Ingestion of FBs in adults can be intentional, as seen in adult patients with intellectual disability and psychiatric diseases. A healthy person may swallow materials (drugs) to smuggle them across borders or airports. Adults may have an external motivation to ingest foreign bodies to avoid jail sentences. Accidental FB ingestion may include accidental swallowing of dentures or swallowing bones while consuming meat products. Our report emphasizes the importance of psychiatric support to these patients, since a large number of patients often perform such actions repeatedly. Our case is unique for many reasons. The FB in the present case was a metallic, non-foldable size 17 open-end/ring spanner (Fig. 5). Swallowing such an FB requires the mouth opening to be aligned with the esophageal opening, which in turn requires full extension of the neck. We removed the FB under general anesthesia and endotracheal intubation with the neck fully extended. Moreover, the FB passed the normal physiological esophageal constrictions. Thus, the patient may have pushed the FB entirely, occupying the entire esophagus by his fingers and lodging the object inside the esophagus (Fig. 1). We searched the literature about FBs impacted in the throat and in the esophagus in adults. To the best of our knowledge, the object in the present case was the largest FB impacted in the esophagus. It was removed without esophageal perforation or injury. Since repeated incidence of such an event can be expected in this patient as part of self-mutilating behavior, prevention of FB ingestion and therapy for the same would require a multidisciplinary approach.

## 4 Conclusion

An FB impacted in the esophagus is an emergency that needs to be attended as soon as possible due to the high risk of morbidity and mortality. Intentional FB ingestion is observed in psychiatric patients as self-harming behavior, as a way of seeking attention of the family, or as a suicide attempt. It may also be observed in prisoners as an attempt to exit from the prison. Close monitoring of psychiatric patients is required to avoid complications associated with the ingestion of FBs. Timely counseling and mental support are essential for proper management of such cases.

## Consent

Taken from the patient and his family.

## Ethical approval

Ethical approval agreed by the ethical committee of the Department of Surgery, College of Medicine, Jabir Ibn Hayyan Medical University.

## Funding

Nil.

## Author contribution

Firas Shaker Mahmoud Al-Faham: Surgeon who performs the intervention, collection of data Samer Makki Mohamed Al-Hakkak: Design of study, data analysis and interpretation and writing the paper.

## Research registration number

Researchregistery 5426.

## Guarantor

Dr Samer Makki Mohamed Al-Hakkak.

## Provenance and peer review

Not commissioned, externally peer reviewed.

## Declaration of competing interest

Nil.
